# Cardiomyocyte-Derived Exosomes: Biological Functions and Potential Therapeutic Implications

**DOI:** 10.3389/fphys.2019.01049

**Published:** 2019-08-20

**Authors:** Hui Yu, Zhanli Wang

**Affiliations:** The Second Affiliated Hospital, Baotou Medical College, Baotou, China

**Keywords:** cardiomyocyte, exosome, biological function, cardiovascular disease, therapeutic application

## Abstract

Exosomes, which are membrane-enclosed nanovesicles released by almost all cell types, have been recognized to play important roles in mediating cell–cell communication. In recent years, the physiological and pathological effects of exosomes on cardiovascular disease have been extensively studied. Exosomes can transfer proteins, mRNAs, microRNAs, and other bioactive molecules to recipient cells to influence their biological properties. In recent years, accumulating evidence has suggested that cardiomyocyte-derived exosomes play an important role in the progression of cardiovascular disease. Here, we summarize the functional roles of cardiomyocyte-derived exosomes in cardiovascular physiology and pathology.

## Introduction

Heart disease, or CVD, has become the most common cause of mortality globally (Lai et al., 2019). Major types of heart disease include coronary artery disease, stroke, heart failure, hypertensive heart disease, rheumatic heart disease, cardiomyopathy, inflammatory heart disease, and others (GBD 2013 Mortality and Causes of Death Collaborators, 2015). The heart consists of a complex mixture of various cell types, including cardiomyocytes, fibroblasts, endocardial and epicardial cells, inflammatory cells, and immune cells. These cells within the heart communicate intensively to facilitate proper cardiac function through direct cell–cell contact and paracrine interactions (Talman and Kivelä, 2018).

Exosomes, which were first identified by Johnstone et al. (1987), are small membrane-bound vesicles with a diameter of 30–100 nm. Exosomes are secreted by most cell types, including lymphocytes, platelets, and adipocytes, as well as tumor, muscle, and stem cells (Brisson et al., 2017; Barlow and Solomon, 2018; Meng et al., 2019; Scherer, 2019; Seo et al., 2019; Torralba et al., 2019). Exosomes mediate intercellular signaling and communication by shuttling nucleic acids, proteins, and lipids between cells; knowledge of their functions can provide novel diagnostic and therapeutic strategies for many diseases (Fruhbeis et al., 2012; You et al., 2018; Wu et al., 2019). Similarly, in the heart, exosomes can act as vehicles to deliver cargo to neighboring or distant cells to reprogram the cardiac microenvironment (Kishore et al., 2016; Juni et al., 2017; Marini et al., 2017; Poe and Knowlton, 2017). Indeed, exosomes play a substantial role in various processes involved in the pathogenesis of CVDs such as cardiac fibrosis, hypertrophy, myocardial apoptosis, and angiogenesis (Bei et al., 2017a; Salem and Fan, 2017; Davidson and Yellon, 2018). In this review, we summarize current knowledge regarding cardiomyocyte-derived exosomes and their applications in the diagnosis and possible repair of cardiac damage.

## Biogenesis of Cardiomyocyte-Derived Exosomes

**FIGURE 1:**
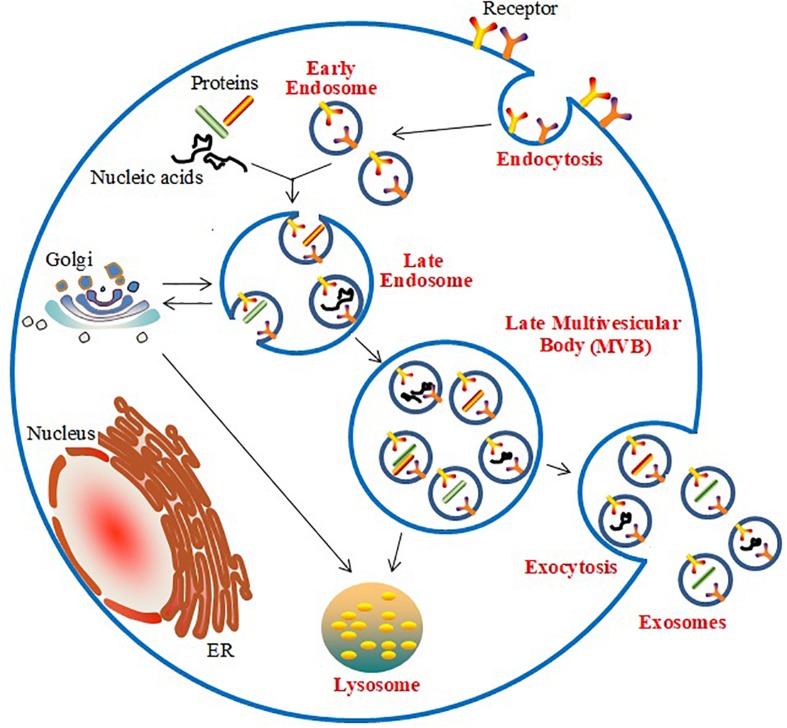
Biogenesis and release of exosomes.

The generation of exosomes is associated with the endosomal network (Stoorvogel et al., 2002; Akers et al., 2013; Frydrychowicz et al., 2015; Figure 1). Initially, the inward budding of the cell membrane forms early endosomes, which are embedded with specific membrane proteins. Subsequently, various ILVs are generated by further inward budding of the endosomal membrane; these early endosomes are known as multi-vesicle bodies (MVBs). Finally, the MVBs fuse with lysosomes and release ILVs for degradation or recycling, or they fuse with the plasma membrane and release the ILVs as exosomes. Endosomal Sorting Complex Required for Transport (ESCRT)-dependent and -independent mechanisms participate in the biogenesis of exosomes in MVBs (Jakhar and Crasta, 2019). The ESCRT-dependent pathway, which includes the distinct subcomplexes ESCRT-0, ESCRT-I, ESCRT-II, and ESCRT-III, directs the sorting of ubiquitinated proteins and is responsible for the inward budding of ILVs into the interior of endosomes, thereby forming MVBs (Kowal et al., 2014). The ESCRT-independent pathway in the biogenesis of exosomes has been found to require the participation of the Golgi apparatus to package proteins into exosomes within MVBs (Trajkovic et al., 2008).

The generation and release of exosomes derived from cardiomyocytes is influenced by many factors. Previous findings showed that Hsp20 mediated the activation of exosome biogenesis and their secretion from cardiomyocytes by interacting with Tsg101, a major exosome biogenesis mediator (Wang et al., 2016). Cardiomyoblasts also display increased exosome excretion under glucose starvation (Garcia et al., 2015). Loyer et al. (2018) demonstrated that myocardial infarction triggers the release of cardiomyocyte-derived exosomes. Additionally, previous reports have demonstrated that ethanol treatment increased exosome formation and secretion from cardiomyocytes (Malik et al., 2013). Furthermore, treatment with TGF-β and PDGF also affected the characteristics of biogenesis and the release of exosomes in cultured cardiomyocytes (Gennebäck et al., 2013). Moreover, under stresses such as increased Ang II production, hypoxia, inflammation, or injury, cardiomyocytes have been found to display increased exosome secretion (Chistiakov et al., 2016; Yang et al., 2018).

## The Reported Molecular Contents of Cardiomyocyte-Derived Exosomes

To date, numerous studies have indicated that exosomes carry an extensive range of biomolecules including proteins, lipids, carbohydrates, mitochondrial DNA (mtDNA), mRNAs, miRNAs, and long non-coding RNAs (lncRNAs). However, the composition of exosomes is influenced by the cell of origin and the pathophysiological status leading to exosome formation, suggesting the potential functional diversity of exosomes (Katsuda et al., 2014; Conigliaro et al., 2017; Cheng et al., 2018). Recently, a large variety of biomolecules have been identified in exosomes secreted from cardiomyocytes in several studies. Expectedly, the presence of proteins within exosomes has been consistently shown in recent reports (Table 1). Hsps play essential roles in cellular survival and adaptation under numerous stresses (Gupta and Knowlton, 2007). An increasing number of observations have shown that cardiomyocyte-derived exosomes are enriched for Hsps (Hsp20, Hsp60, and Hsp70) involved in regulating cardiomyocyte growth and survival under stress (Gupta and Knowlton, 2007; Zhang et al., 2012; Malik et al., 2013; Feng et al., 2014; Yu et al., 2019). Recent findings have also demonstrated that myocyte-derived exosomes contain inflammatory factors such as IL-6 and TNF-α, which are responsible for cardiac remodeling (Yu et al., 2012; Datta et al., 2017). Furthermore, exosomes derived from cardiomyocytes were found to carry functional GLUT (GLUT4, GLUT1) and glycolytic enzymes (lactate dehydrogenase), and were shown to have specialized functions in glucose transport and metabolism in endothelial cells (Garcia et al., 2016). Malik et al. (2013) investigated the protein contents of different exosome extracts after ethanol or hypoxia/reoxygenation treatment by mass spectrometry. They identified 51 different proteins in exosomes derived from ethanol-treated cells, and 33 proteins in those derived from hypoxia/reoxygenation-treated cells, ranging from membrane-bound to cytosolic and mitochondrial proteins.

**TABLE 1:** The reported proteins within cardiomyocyte-derived exosomes.

**Exosomal cargo**	**Expression**	**Biological function**	**References**
Hsp20	Increased	Promote angiogenesis; Improve cardiac function	Zhang et al., 2012; Yu et al., 2019
Hsp60	Increased	Promote immune responses	Gupta and Knowlton, 2007
Hsp70	Increased	Improve cardiac function	Feng et al., 2014
TNF-α	Increased	Contribute to cardiac remodeling	Yu et al., 2012
IL-6	Increased	Contribute to cardiac fibroblasts	Datta et al., 2017
GLUT1	Increased	Regulate metabolism	Garcia et al., 2016
GLUT4	Increased	Regulate metabolism	Garcia et al., 2016

Many studies have demonstrated that exosomes derived from cardiomyocytes can also carry nucleic acid cargo. Waldenström et al. (2012) identified 343 different sequences of chromosomal DNA in microvesicles/exosomes derived from the cultured cardiomyocytes. Wang et al. (2014) reported that exosomes secreted from the cardiomyocytes of diabetic patients carried higher levels of miR-320 and lower levels of miR-126 compared to those secreted from healthy cardiomyocyte-derived exosomes. Exosomal miR-320 exerts an anti-angiogenic function by suppressing its target genes in recipient endothelial cells, including Hsp20, V-ets erythroblastosis virus E26 oncogene homolog 2 (Ets2), and IGF-1. Recent studies also indicated that miR-30a was present at high levels in hypoxic cardiomyocyte-derived exosomes (Yang et al., 2016). Chaturvedi et al. (2015) further found that exosomes from cardiomyocytes were enriched for certain miRNAs (particularly miR-29b, miR-323-5p, miR-455, and miR-466) and mediated the regulation of MMP9 expression in diabetic heart tissues. Additionally, miR-27a, miR-28-3p, miR-34a, and miR-208a were found to be highly expressed in cardiomyocytes and preferentially incorporated into exosomes (Tian et al., 2018; Yang et al., 2018). Table 2 shows the major reported cardiomyocyte-derived exosomal miRNAs and their biological functions. As carriers of various biomolecules, exosomes protect their cargo against digestion by enzymes present in the body fluid. Therefore, the encapsulated contents can be transported to their extracellular destinations (Katsuda et al., 2014; Ohno and Kuroda, 2016).

**TABLE 2:** The reported miRNAs within cardiomyocyte-derived exosomes.

**Exosomal cargo**	**Expression**	**Biological function**	**References**
miR-320	Increased	Inhibit angiogenesis	Wang et al., 2014
miR-30a	Increased	Regulate autophagy	Yang et al., 2016
miR-29b	Increased	Inhibit fibrosis and myocyte uncoupling	Chaturvedi et al., 2015
miR-455	Increased	Inhibit fibrosis and myocyte uncoupling	Chaturvedi et al., 2015
miR-27a	Increased	Contribute to oxidative stress	Tian et al., 2018
miR-28-3p	Increased	Contribute to oxidative stress	Tian et al., 2018
miR-34a	Increased	Contribute to oxidative stress	Tian et al., 2018
miR-208a	Increased	Promote fibroblast proliferation	Yang et al., 2018

## Isolation of Cardiomyocyte-Derived Exosomes

Exosome isolation is a fast-growing field of study. The efficient isolation of high-quality exosomes is critical for determining their applications in biomedical sciences. However, since exosomes are very small vesicles, there are several technical issues impeding their successful isolation (Gurunathan et al., 2019). One challenge is the absence of suitable techniques for accurate exosome characterization. Currently, various techniques for the isolation of exosomes have been reported, such as ultracentrifugation (Van Deun et al., 2014), size-based filtration (Zeringer et al., 2015), size-exclusion chromatography (Momen-Heravi et al., 2012), polymer precipitation (Ibrahim et al., 2014), immuno-affinity purification (Zarovni et al., 2015), and microfluidics-based isolation techniques (Wang et al., 2013). Each approach has its disadvantages and advantages. Ultracentrifugation at high speeds can be used to obtain a highly pure exosomal fraction from cell culture media. However, this method is not suitable for small volumes of clinical samples with complex mixtures containing a large number of components, because it is difficult to remove other contaminating membrane vesicle populations using this method (Witwer et al., 2013). Size-based filtration methods are not suitable for the enrichment of exosomes (Gurunathan et al., 2019). It must be emphasized that size exclusion chromatography and immuno-affinity purification cannot be used to discriminate larger fragmented microparticles from exosomes, leading to the impurity of yields (György et al., 2011; Peterson et al., 2015). Recent studies have suggested that immune affinity capture is a specific technique to isolate exosomes; however, yields are generally low from this method (Prunotto et al., 2013). In addition to these traditional isolation techniques, many commercial kits for the isolation of exosomes are available, such as ExoQuick Exosome Precipitation Solution, Total Exosome Isolation, and exoRNeasy Serum/Plasma Kit (Macías et al., 2019). ExoQuick Exosome Precipitation Solution and Total Exosome Isolation are convenient precipitation solutions, which have been utilized to precipitate particles in liquid, but they are unable to resolve particle heterogeneity (Lobb et al., 2015). The exoRNeasy Serum/Plasma kit was used for isolation of exosomes using a membrane-based affinity binding step from serum or plasma (Enderle et al., 2015). Recently, Malik et al. (2016) reported a simple, low-cost, and effective method for the isolation of exosomes derived from cardiac myocytes. In brief, cardiac myocytes are cultured in Media 199 for 2 h, and the medium is then replaced with albumin-free Media 199. Next, cardiac myocytes are treated with cell culture-grade ethanol or with hypoxia-inducing factors. After treatment, the media is collected and centrifuged at 300 × *g* for 10 min, followed by centrifugation at 4,700 × *g* for 30 min. The resulting supernatant is then collected and concentrated. Next, ExoQuick is used to complete the isolation of the exosomes. Their study further evaluated the reliability and effectiveness of this approach in terms of the purity, size, morphology, and proteome content of extracted exosomes. Their paper further confirmed the high quality and reliability of this method, which was shown to be efficient, reliable, and reproducible. However, this approach for the purification of cardiac myocyte exosomes is limited to conditioned media. The water, tubing and ethanol used for the cardiac myocytes isolation must be cell culture grade, avoiding the cardiac myocytes damage. It is also very important to perfuse the isolated heart in order to obtain as many living cells as possible. Moreover, the adult cardiac myocytes should be treated with ethanol to increase exosome production and the suitable ethanol concentration was very important.

Moreover, researchers rely on the identification of certain exosomal markers to confirm the presence of exosomes (Mathivanan and Simpson, 2009). The tetraspanin CD63 and tumor susceptibility gene protein 101 (TSG101) are common exosomal marker proteins found in cell suspension (Gennebäck et al., 2013). The tetraspanins CD9 and CD81 are also molecular markers of cardiomyocyte-derived exosomes (Wang et al., 2014; Garcia et al., 2015; Yang et al., 2018). Moreover, Hsp70 is a specific marker of cardiomyocyte-derived exosomes, and can be identified by western blotting (Kuo et al., 2019). However, exosomes formed through ESCRT-independent pathways are usually devoid of biomarkers associated with the ESCRT complex, such as CD63 or CD9 (Akers et al., 2013). To rule out the contamination of other membrane vesicle populations, researchers have often performed the simultaneous detection of endoplasmic reticulum-related markers such as calnexin and glucose-regulated protein 78 (Grp78) (Gennebäck et al., 2013; Kuo et al., 2019).

## Cardiomyocyte-Derived Exosomes and Cardiovascular Cell–Cell Communication

**FIGURE 2:**
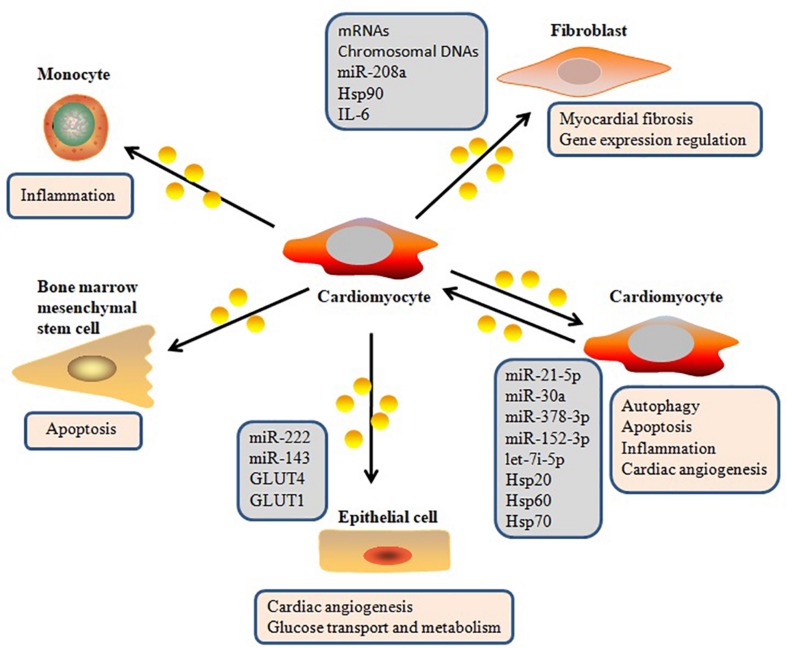
Exosome-mediated cell–cell communication in heart.

In addition to other extracellular molecules that can mediate intercellular communication, increasing attention has been given to exosomes (Pegtel et al., 2010; Cervio et al., 2015). Exosomes have become an important player in intercellular signaling via several types of interaction pathways, including delivering their molecular cargo into the target cell, directly regulating membrane receptors of the target cell, and changing the microenvironmental milieu of the target cell (Sluijter et al., 2014; Poe and Knowlton, 2018; Figure 2). Waldenström et al. (2012) demonstrated that cardiomyocyte-derived exosomes packaged DNA-based messages into recipient fibroblasts and are involved in various cell-related processes in the recipient fibroblasts by regulating gene expression (Waldenström et al., 2012). MiR-30a, which is derived from hypoxic cardiomyocytes, is efficiently transferred between cardiomyocytes via exosomes, and regulates autophagy by affecting the expression of Beclin-1, ATG12, and the ratio of LC3II/LC3I, which are important regulators of autophagy (Yang et al., 2016). Yang et al. (2018) also confirmed that cardiomyocyte-derived exosomes can be absorbed by fibroblasts, and that these exosomes transfer miR-208a between myocytes and fibroblasts. Their data suggested that crosstalk between myocytes and fibroblasts via exosomes contributed to the development of myocardial fibrosis. Recently, it was reported that injured cardiomyocyte-derived exosomes accelerated the injury of bone marrow-derived mesenchymal stem cells transplanted into a heart infarction mouse model by mediating cell–cell communication (Hu et al., 2018). Recent research has described the molecular mechanisms of the intracellular autophagy pathway induced by exosomes, which delivers autophagy-associated molecules (Zheng et al., 2019). Bollini et al. (2018) reported that exosomes play significant roles in intercellular signaling, and that cardiomyocyte-derived exosomes affect the function of other cardiac cell types, thereby influencing many physiological and pathological functions of the heart. Yuan et al. (2016) also reported that exosomes from cardiomyocytes mediated cardiac repair after myocardial infarction by delivering a variety of functional molecules into their target cells. Ribeiro et al. (2013) further found that exosomes secreted by cardiomyocytes can carry and transfer pro-angiogenic and anti-angiogenic factors, indicating that they play a role in regulating angiogenesis. Taken together, recent evidence has established that exosomes secreted from cardiomyocytes can deliver a wide variety of biomolecules into other cell types and regulate gene expression in these cells.

It should be noted that the presence and relative abundance of biomolecules inside or on the surface of exosomes can fluctuate depending on the cell type and pathophysiological state (Zhou et al., 2006; Cosme et al., 2017; da Silva Novaes et al., 2019). Recently, Kuo et al. (2019) reported that simvastatin, a potent competitive inhibitor of 5-hydroxy-3-methylglutaryl-coenzyme A reductase, attenuated collagen-associated protein expression in cardiomyocyte-derived exosomes and reduced the uptake of the exosomes by human cardiac fibroblasts. Yang et al. (2018) also found that, in a rat model of cardiac fibrosis, miR-208a levels were up-regulated in cardiomyocyte-derived exosomes. The onset of myocardial infarction also induced the release of miRNA-enriched exosomes (Abreu and da Costa Martins, 2018). Additionally, diabetic cardiomyocytes exhibited increased secretion of detrimental exosomes containing decreased Hsp20 levels, which contributed to diabetes-induced organ damage (Wang et al., 2016).

## Potential Diagnostic and Therapeutic Applications of Cardiomyocyte-Derived Exosomes

Emerging evidence shows that exosomes have great diagnostic and therapeutic potential (Cobelli et al., 2017; Kim and Kim, 2017; Campanella et al., 2019; Urban et al., 2019). The remarkable diagnostic and therapeutic potential of exosomes in CVDs has been increasingly investigated in recent years (Bei et al., 2017b; Sun et al., 2017; Ghafarian et al., 2018). A large number of studies have confirmed the diagnostic and therapeutic applications of exosomes derived from cardiac fibroblasts, cardiac telocytes, cardiosphere-derived cells, and cardiac progenitor and stem cells in terms of CVD (Bang et al., 2014; Barile et al., 2014; Fertig et al., 2014; Khan et al., 2015; Lyu et al., 2015; Kervadec et al., 2016; Xiao et al., 2016; Agarwal et al., 2017; Gallet et al., 2017; Barile et al., 2018; Gao et al., 2018; Ju et al., 2018; Namazi et al., 2018; Zhu et al., 2018). Recent findings have confirmed the important role of cardiomyocyte-derived exosomes in the diagnosis, prognosis, and therapy of various diseases. Exosomes secreted from dystrophin-deficiency-induced pluripotent stem cell-derived cardiomyocytes exert cardioprotection via the presence of exosomal surface proteins and the activation of ERK1/2 and p38MAPK signaling (Gartz et al., 2018). Upon myocardial infarction, cardiomyocyte-derived exosomes were shown to regulate local inflammatory responses through the stimulation of cardiac monocytes (Loyer et al., 2018). Therefore, it is thought that the exosome is an important component of the cardiac microenvironment and plays a complex role in the treatment of myocardial infarction. Similarly, exosomes secreted from injured cardiomyocytes were found to exert negative effects similar to those of cardiomyocyte-derived exosomes in myocardial infarction (Hu et al., 2018). Cardiomyocyte-derived exosomes also mediate intercellular communication for myocardial repair and regeneration (Ong et al., 2018). Studies have demonstrated that dysregulated miRNAs contained in cardiomyocyte-derived exosomes, such as let-7i-5p, miR-21-5p, miR-27a, miR-28-3p, miR-34a, miR-143, miR-222, and miR-378-3p, regulate cardiac function by targeting different mRNAs (Ribeiro-Rodrigues et al., 2017; Zhang et al., 2017; Tian et al., 2018). These findings will accelerate the development of promising therapeutic strategies for patients with ischemic heart disease and chronic heart failure. Further studies are urgently needed to explore the possible molecular mechanisms underlying the selective loading and accumulation of certain molecules, substrate specificity, and the regulation of target cell by exosomes (Jansen and Li, 2017; Vanhaverbeke et al., 2017).

## Conclusion and Future Perspectives

The field of exosome analysis has expanded greatly in recent years. In this review, we concentrated on the biological functions and potential therapeutic implications of cardiomyocyte-derived exosomes, including biogenesis, molecular contents, isolation techniques, biomarkers, cardiovascular cell–cell communication, and potential diagnostic and therapeutic applications of various diseases.

Although exosome biology represents a fascinating area for future therapeutic cardioprotection strategies, there remain several technical challenges to this field of research. The current major technical challenge is the ability to differentiate exosomes derived from cells under normal and pathological conditions for therapeutic applications. Another challenge is the application of exosomal research methodologies and tools *in vivo*. Once these challenges are overcome, new techniques for the efficient isolation, quantification, and analysis of exosomes will be helpful to elucidate the function and influence of exosomes in the cardiac microenvironment *in vivo*, and will facilitate the development of new therapeutic strategies to efficiently diagnose and treat heart diseases.

## Author Contributions

Both authors listed have made a substantial, direct and intellectual contribution to the work, and approved it for publication.

## Conflict of Interest Statement

The authors declare that the research was conducted in the absence of any commercial or financial relationships that could be construed as a potential conflict of interest.

## Abbreviations

Ang II: Angiotensin II
ATG12: autophagy-related gene 12
BMSCs: bone marrow mesenchymal stem cells
CCL: chemokine ligand
CVD: cardiovascular disease
ERK1/2: extracellular signal-regulated kinase 1/2
ESCRT: endosomal sorting complexes required for transport
GLUT: glucose transporters
Hsp: heat shock protein
IGF-1: insulin-like growth factor-1
ILV: intraluminal vesicle
IL-6: interleukin-6
LC3: microtubule associated protein light chain 3
miRNA: microRNA
MMP9: matrix metalloprotein 9
mRNA: messenger RNA
mTOR: mammalian target of rapamycin
MVB: multivesicle body
Nrf2: nuclear factor erythroid-2 related factor 2
PDGF: platelet-derived growth factor
p38 MAPK: p38 mitogen-activated protein kinase
TGF- β: transforming growth factor beta
TNF- α: tumor necrosis factor- α.
